# The Anti-Inflammatory Effects of Blueberries in an Animal Model of Post-Traumatic Stress Disorder (PTSD)

**DOI:** 10.1371/journal.pone.0160923

**Published:** 2016-09-07

**Authors:** Philip J. Ebenezer, C. Brad Wilson, Leslie D. Wilson, Anand R. Nair, Francis J

**Affiliations:** 1 Comparative Biomedical Sciences, School of Veterinary Medicine, Louisiana State University Baton Rouge, Louisiana, United States of America; 2 Pathobiological Sciences, School of Veterinary Medicine, Louisiana State University Baton Rouge, Louisiana, United States of America; Universidade de Sao Paulo, BRAZIL

## Abstract

Post-traumatic stress disorder (PTSD) is a trauma and stressor-related disorder that results in a prolonged stress response. It is associated with increased oxidative stress and inflammation in the prefrontal cortex (PFC) and hippocampus (HC). The only approved therapy for PTSD is selective serotonin re-uptake inhibitors (SSRIs), but their efficacy is marginal. Recently, we demonstrated that over-production of norepinephrine (NE) as the possible reason for the lack of efficacy of SSRIs. Hence, there is a need for novel therapeutic approaches for the treatment of PTSD. In this study, we investigated the anti-inflammatory role of blueberries in modulating inflammatory markers and neurotransmitter levels in PTSD. Rats were fed either a blueberry enriched (2%) or a control diet. Rats were exposed to cats for one hour on days 1 and 11 of a 31-day schedule to simulate traumatic conditions. The rats were also subjected to psychosocial stress via daily cage cohort changes. At the end of the study, the rats were euthanized and the PFC and HC were isolated. Monoamines were measured by high-performance liquid chromatography. Reactive oxygen species (ROS), gene and protein expression levels of inflammatory cytokines were also measured. In our PTSD model, NE levels were increased and 5-HT levels were decreased when compared to control. In contrast, a blueberry enriched diet increased 5-HT without affecting NE levels. The rate limiting enzymes tyrosine hydroxylase and tryptophan hydroxylase were also studied and they confirmed our findings. The enhanced levels free radicals, gene and protein expression of inflammatory cytokines seen in the PTSD group were normalized with a blueberry enriched diet. Decreased anxiety in this group was shown by improved performance on the elevated plus-maze. These findings indicate blueberries can attenuate oxidative stress and inflammation and restore neurotransmitter imbalances in a rat model of PTSD.

## Introduction

Post-traumatic stress disorder (PTSD) is a trauma and stressor-related disorder that results in a prolonged stress response [1, 2]. Although PTSD is a psychiatric disorder and has been classified as such for 35 years, the Diagnostic and Statistical Manual of Mental Disorders Fifth Edition, (DSM-V) re-categorized it to a trauma and stressor-related disorder, with a lifetime prevalence rates of 6.8% on men in the US and making it one of the most common psychiatric illness. It also known as delayed psychogenic reaction, which develops after a life-threatening traumatic incident such as bombing on the battlefield, natural disasters may or may not involve physical injury, or the danger of physical detriment. It was defined in 1980, partly based on the experiences of soldiers and victims of war [3]. It is the 4^th^ most common psychiatric disorder, with a lifetime prevalence rate of 6.8% in the US [4]. The clinical manifestations of PTSD are characterized by intense fear, helplessness and horror following repeated experience of flashback memories and leads to elusive behavior [5, 6]. In the past decade extraordinary progress has been made to understand the pathogenesis of PTSD, but it is not yet completely clear [7]. The stressful event can alter brain chemistry [1] and modulate the generation of reactive oxygen species (ROS), protein [8] and gene expression of inflammatory cytokines and neurotransmitters [9–12]. These molecules play a significant role in the progression of PTSD and might be potential targets for pharmacologic intervention. A wide range of pharmacological therapeutics is available to treat some of the factors associated with PTSD, but these medicinal agents have side effects. Hence, there is a need for a novel therapeutic and nonpharmacological approach for the treatment of PTSD.

Flavonoids, a major subclass of polyphenols, can be found in a variety of foods. Blueberries contain a significant amount of flavanoids, specifically anthocyanins, which are well known for their anti-inflammatory and antioxidant properties [13–17]. Recent studies show that Blueberries (*Vaccinium sp*.) may beneficially modulate the immunological profile distinct from inhibiting pro-inflammatory molecules [18–22]. In the present study the free radical scavenging ability of blueberries was evaluated by the quantitative analysis of free radicals, neurotransmitters, protein and gene expression of inflammatory cytokines in a PTSD inducing rat model.

Free radicals including ROS participate in the control of normal cell functions [23], but inappropriate amounts of ROS damage tissues [12]. Most investigators agree that ROS is one of the fundamental causes of inflammation associated oxidative stress in PTSD [24]. There is increasing experimental evidence indicating the impairment of cellular total antioxidant capacity in PTSD [25]. The significant increase in the generation of ROS in PTSD lead to perturbation of brain cells and ends in inflammation [10, 11, 24]. These abnormal changes interrupt cellular functions such as proliferation, differentiation, transformation and apoptosis through different signaling cellular pathways.

Neurotransmitters play a significant role in the regulation of brain functions such as attention, memory, mood, behavior and autonomic control [9]. These functions are significantly compromised in PTSD due to a loss of balance in neurotransmitters, which regulate the cognitive brain functions [26]. The biogenic amines such as 5-hydroxytryptamine (5-HT) and norepinephrine (NE) have been identified as the principal neurotransmitters mediating fast excitatory synaptic responses in the central nervous system (CNS) and have widespread influence on the prefrontal cortex, basal ganglia, limbic areas and spinal cord [27]. The neocortex and hippocampus are susceptible to inflammation in response to traumatic conditions that lead to disruption of the neuronal circuitry [28, 29].

Toll-like receptor 4 (TLR4) is a protein encoded by the TLR4 gene and plays a key role in the innate immune system. Earlier studies show that TLR4 induced the cytokine generation and expression of cofactors through NF-κB signaling pathway [30]. High-mobility group protein B1, also known as high-mobility group protein 1 (HMG1), is a protein which is encoded by the HMGB1 gene [31]. The inflammation associated with oxidative stress in PTSD alters the protein and gene expression of inflammatory cytokines. Our lab recently reported that inflammatory cytokines and ROS were elevated in the brain and systemic circulation during PTSD like inflammation associated with oxidative stress development [9–12]. In addition, our recent findings show that the over-activation of NE as the possible reason for the lack of efficacy of the selective serotonin reuptake inhibitor (SSRI) sertraline, one of the approved medications for PTSD [10].

Since blueberries have been shown to attenuate oxidative stress and inflammation [32], we examined the anti-inflammatory effects of blueberries against detrimental effects of PTSD like inflammation associated with oxidative stress in the brain. In this study, we examined whether a blueberry enriched diet could modulate redox imbalances, neurotransmitters and behavior in response to a predator exposure / psychosocial stress rodent model of pre-clinical PTSD.

## Materials and Methods

### Ethics statement

This study was carried out in strict accordance with the recommendations in the Guide for the Care and Use of Laboratory Animals of the National Institutes of Health. The protocol was approved by the Committee on the Ethics of Animal Experiments of the Louisiana State University.

### Animals and experimental Design

**Fig 1:**
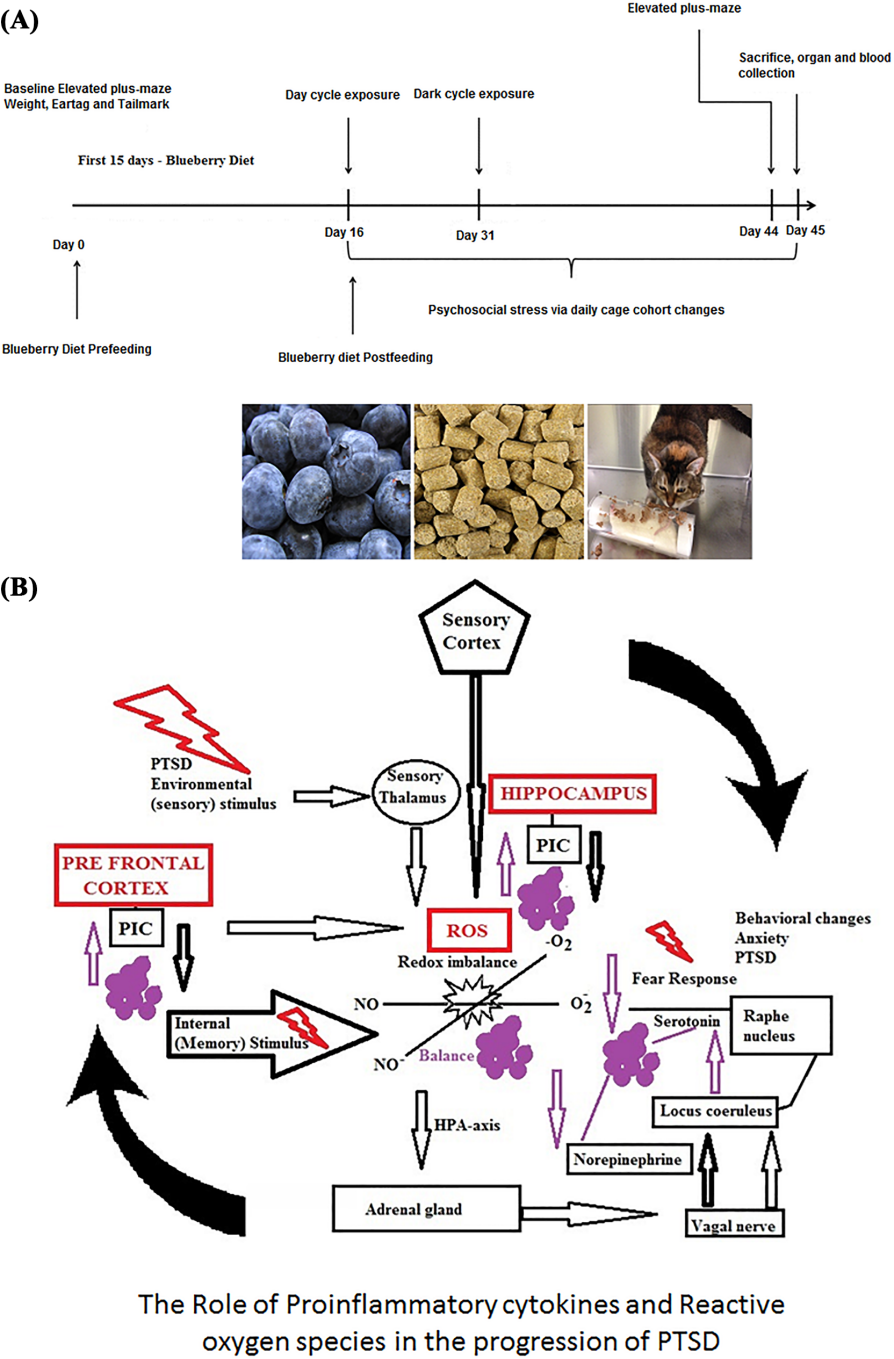
a–Scheme: The predator exposure/psychosocial stress model includes two cat exposures over a 45 and 31 day period, combined with daily cage cohort changes. The experimental animals were fed with 2% blueberry diet 15 days ahead of the cat exposure (Total 45 days–blueberry diet pre feeding group) and blueberry diet post feeding group along with first cat exposure (Total 31 days). The Anxiety levels were measured at the baseline and at the end of the stress schedule via Elevated plus maze. b—The role of proinflammatory cytokines and reactive oxygen species in the progression of PTSD.

Naive adult male Sprague-Dawley rats (Harlan Laboratories, Indianapolis, IN) were used in all experiments (n = 12). The rats were the same age (10 weeks) and approximately the same weight (± 15 g) upon delivery. Rats were pair-housed in standard plastic cages with access to food and water *ad libitum*. The cages were maintained in ventilated racks and randomly assigned to a rack location to ensure groups were evenly distributed. The vivarium room was kept on a 12-h light/dark cycle (0700–1900), temperature was maintained at 20 ± 1°C, and humidity ranged from 23–42%. After a 1-week acclimation period, the mean weight of all rats was 335.01 g ± 3.02 g. Two cats, (Harlan Laboratories, Indianapolis, IN) were used for all predator exposures. They were housed in an open room (15′ × 15′) in the vivarium with access to food, water, and enrichment devices *ad libitum*. The cat room was on the equivalent light/dark cycle and maintained at a similar temperature and humidity. The food consumption (Diets: 2% Blueberry diet (2016 –TD.10445) was measured daily for a week prior to the start of the study. The freeze–dried powder (40513) was obtained from the United States Highbush Blueberry Council (USHBC), and is a blend of two varieties, Tifblue and Rubel. To prepare the 2% blueberry diet, the freeze-dried powder was added to the rodent chow (Harlan TD 10445 2% blueberry diet– 2016).”The amount of corn in the control diet was adjusted to compensate for the added volume of blueberries, in order to make the two diets isocaloric [22]. Food consumption was measured weekly for the chronic feeding studies by weighing feed before placing it in each cage, and subtracting the weight of remaining feed at the end of each week (Fig 1A and 1B).

### Stress Induction

The predator exposure/psychosocial stress regimen were performed according to stress regimen, established by Zoladz et al [5] and published and validated by our lab [9–12]. It was designed to induce a PTSD-like syndrome as true PTSD is clinically defined as a human disorder designed to produce a pre-clinical PTSD that closely mimics signs and symptoms seen in human patients [5]. PTSD rats were individually isolated in cylindrical, Plexiglas containers (IITC Life Science, Inc., Woodland Hills, CA; tail cuff restraint containers for 400–600 g rats and Kent Scientific, Torrington, CT; tail cuff restraint containers for 300–500 g rats) and canned cat food (Friskies, Purina, St. Louis, MO) was smeared on the outside of the cylinders. The cylinders prevented direct contact with the cats, and the cat food induced predatory movement in the cats. We have performed two cat exposures. The first cat exposure was conducted during the light cycle (0700–1900) for an hour. Ten days later, a second cat exposure was conducted during the dark cycle (1900–0700) for an hour. In addition to the cat exposures, starting on day one the rats were subjected to psychosocial stress by changing their cage cohort daily. The cage cohort rotation was established prior to the start of the experiment, whereby each rat was never housed with the same rat on consecutive days and never housed with the same rat more than four times in a month. The predator exposure/psychosocial stress regimen was continued for 31 days. After 31 days, PTSD and control group rats were euthanized via carbon dioxide inhalation, perfused with a phosphate buffered vascular rinse solution, and the brains were removed. The hippocampus and pre-frontal cortex were dissected and flash-frozen in liquid nitrogen.

Electron paramagnetic resonance (EPR) spectroscopy: Measurement of ROS in prefrontal cortex and Hippocampus

Total ROS were measured in brain tissue hippocampus and PFC via electron paramagnetic resonance (EPR) as previously described [11]. Total ROS levels were compared as repeated measures within the experimental animal groups and also between groups to analyze oxidative stress during PTSD progression and the attenuating effects of the blueberry diet. Spin probe 1-Hydroxy-3-methoxycarbonyl-2,2,5,5- tetramethyl-pyrrolidine (CMH) was used to measure the total ROS. Briefly, pieces of tissue were incubated at 37°C for 30 minutes for ROS measurement; then CMH (200 mM) were added and incubated for an additional 30 minutes. Aliquots of incubated probe media were then taken in 50ul disposable glass capillary tubes (Noxygen Science Transfer and Diagnostics) for the determination of total ROS production. All EPR measurements were performed using an EMX ESR eScan BenchTop spectrometer and super-high quality factor microwave cavity (Bruker Company, Germany).

Elevated plus-maze:

The elevated plus maze was performed on our experimental animals according to our earlier reports [10–12]. Briefly, The experimental animals were placed in the center of the elevated plus-maze (EPM) (EB-Instruments (Bioseb), Tampa Bay, FL) facing an open arm and allowed to roam freely for 300 seconds (5 minutes). Movement was monitored via an overhead camera and captured with a specifically designed software program (BioEPM3C, EB-Instruments, Tampa Bay, FL). The primary measurements were the number of entries into each arm (total ambulations) and the total time spent in the open vs. closed arms. An arm entry was defined as all four feet crossing into a different arm. The stand for the EPM was approximately 36” above the floor, and each arm measured 11 cm X 650 cm.

### High-Performance Liquid Chromatography (HPLC)

#### HPLC preparation of standard solution

Neurotransmitter concentrations were detected using Eicom HTEC 500 high performance liquid chromatography system according to our earlier report [9–11]. Briefly, The standard solutions of norepinephrine (NE; MW 337.3), and isoproterenol (internal standard; MW 247.7), each 1 ng/mL concentration, were prepared by serial dilution in 0.1 M hydrochloric acid including 1 mg/mL EDTA. 5-Hydroxytryptamine (5-HT; MW 212.68) was dissolved in 0.1 M acetic acid including 1 mg/mL EDTA. The standard solutions were prepared and filtered through a 0.45 um centrifuge tube filter before injection into the HPLC system. Different concentrations were injected by maintaining the volume of injection at 10ul in order to quantify sample values after authenticating the retention time of individual neurotransmitters.

#### HPLC-detection of neurotransmitters

HPLC system working conditions: isocratic elution; mobile phase (citrate buffer in methanol with EDTA and sodium octane sulfonate); Eicompack SC-ODS (ID 3.0 X 100mm) column; flow rate 340ul/min; graphite working electrode WE-3G (Gasket GS-25), (+750mV vs. Ag/AgCl electrode); temperature 25°C.

#### HPLC-mobile phase

Citric acid monohydrate (8.84g; MW210.14), and 3.10g of sodium acetate (MW82.03) in 800 ml of MilliQ Ultrapure fresh water (>18.2M2/cm) and 200 ml of HPLC grade methanol were added. EDTA (MW372.24; 0.005g) and sodium octane sulfonate (0.220 g), both from Dojindo Laboratories, Rockville, MD, were added.

### Western Blot Analysis

Tissue homogenates from the hippocampus and PFC were subjected to Western Blot (WB) analysis for the determination of protein levels. The extraction of protein and WB was performed as previously described [9–12]. The primary antibodies IL1b, IL 4, IL-18, TLR4, HMGB 1 and GAPDH were commercially obtained from Abcam, Cambridge, MA. Secondary antibodies were obtained: anti-mouse, 1:5000 dilution and anti-rabbit, 1:5000 dilution (SC-2314 and SC-2004 respectively, Santa Cruz Biotechnology, Santa Cruz, CA). The Immunoreactive bands were visualized using enhanced chemiluminescence (ECL Plus, Amersham), band intensities were quantified using ImageJ imaging software (NIH), and were normalized with GAPDH.

### Real-Time PCR Analysis

Semi-quantitative real-time RT-PCR (n = 6/group) was used to determine the mRNA levels of IL1b, IL 4, IL 18, TLR4, HMGB 1and 18S in the PFC and hippocampus regions of the experimental animals. Briefly, the experimental animals were euthanized using Carbon dioxide inhalation, perfused with a phosphate buffered solution directly into the left ventricle, and the brains were quickly removed, dissected, and immediately flash-frozen in liquid nitrogen. Total RNA isolation, cDNA synthesis and RT-PCR were performed as previously described [20]. Gene expression was measured by the ΔΔCT method and was normalized to GAPDH mRNA levels. The data is presented as fold change of the gene of interest relative to that of control animals.

### Statistical Analysis

All data are presented as mean ± SEM. Statistical analysis was done by one-way ANOVA with a Tukey’s post hoc test for multiple comparisons, and unpaired Student’s T-tests were used for two-column analyses. P-values less than 0.05 were considered statistically significant. Statistical analyses were performed using Prism (GraphPad Software, Inc; version 5.0).

## Results

### Oxidative stress analysis

**Fig 2:**
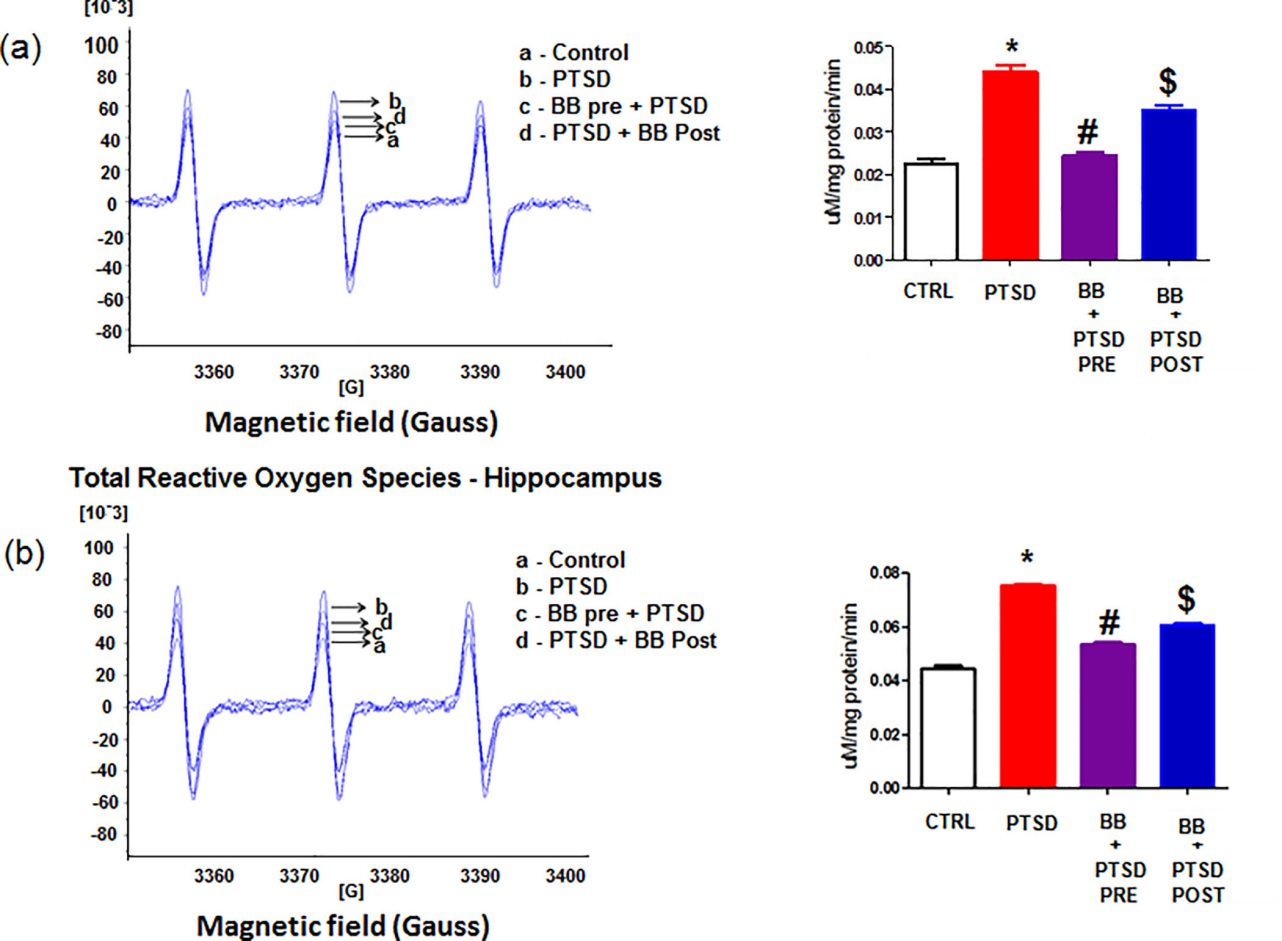
a and b: Reactive oxygen species in the prefrontal cortex and Hippocampus: The total reactive oxygen species were significantly elevated in the PTSD group. The elevation was normalized to control group levels with blueberry diet. Statistical analysis was performed using one way ANOVA with repeated measurements followed by Tukey’s multiple comparison test. *p<0.05 Control Vs PTSD, #p<0.05 PTSD Vs BB = PTSD, and $p<0.05 PTSD Vs PTSD + BB. All data are presented as mean ± SEM. P<0.05 compared to control.

Analysis of EPR data revealed total reactive oxygen species was significantly elevated in the prefrontal cortex and hippocampus in the PTSD group when compared with controls, and it significantly attenuated in the animals fed with 2% blueberry diet (p < 0.05). The group with pre blueberry diet + PTSD Vs PTSD showed more effect than post blueberry diet + PTSD (p < 0.05) (Fig 2A and 2B) when compared with control.

### Elevated plus-maze performance

**Fig 3:**
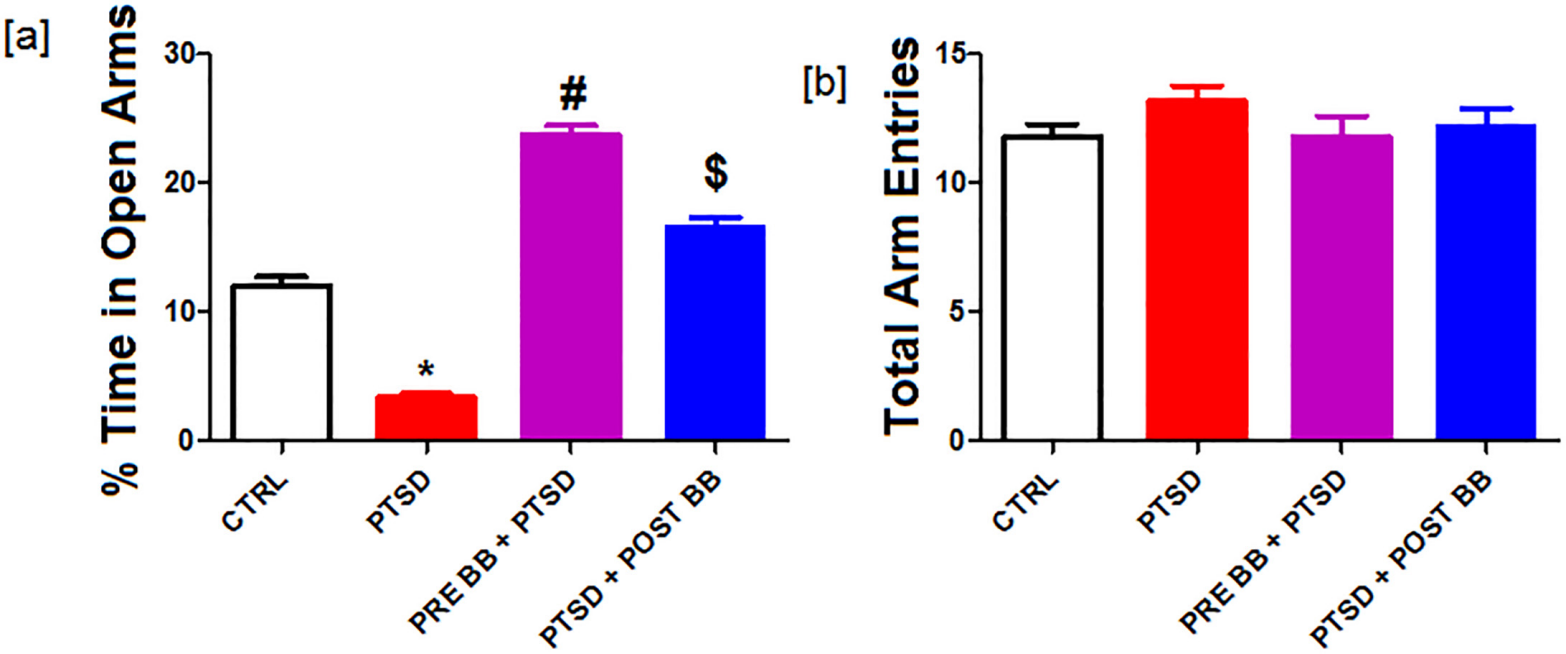
Elevated plus-maze performance: The PTSD group spent significantly less time in the open arms when compared to closed arms, as demonstrated by their reluctance to spend time in the open arms of the elevated plus maze. There were no differences in the total arm entries between groups. Statistical analysis was performed using one way ANOVA with repeated measurements followed by Tukey’s multiple comparison test. *p<0.05 Control Vs PTSD, #p<0.05 PTSD Vs BB = PTSD, and $p<0.05 PTSD Vs PTSD + BB. All data are presented as mean ± SEM. P<0.05 compared to control.

We performed the baseline earlier to the start of the cat exposure; there were no differences in the open arm exploration or total arm entries. After the PTSD stress induction, the PTSD group animals spent less time in the open arms when compared to the closed arms. There were no differences in the total arm entries between groups (p < 0.05). The group with pre blueberry diet + PTSD Vs PTSD spent more time when compared to post blueberry diet + PTSD (p < 0.05) group (Fig 3) when compared with control.

### Neurotransmitter modulation

**Fig 4:**
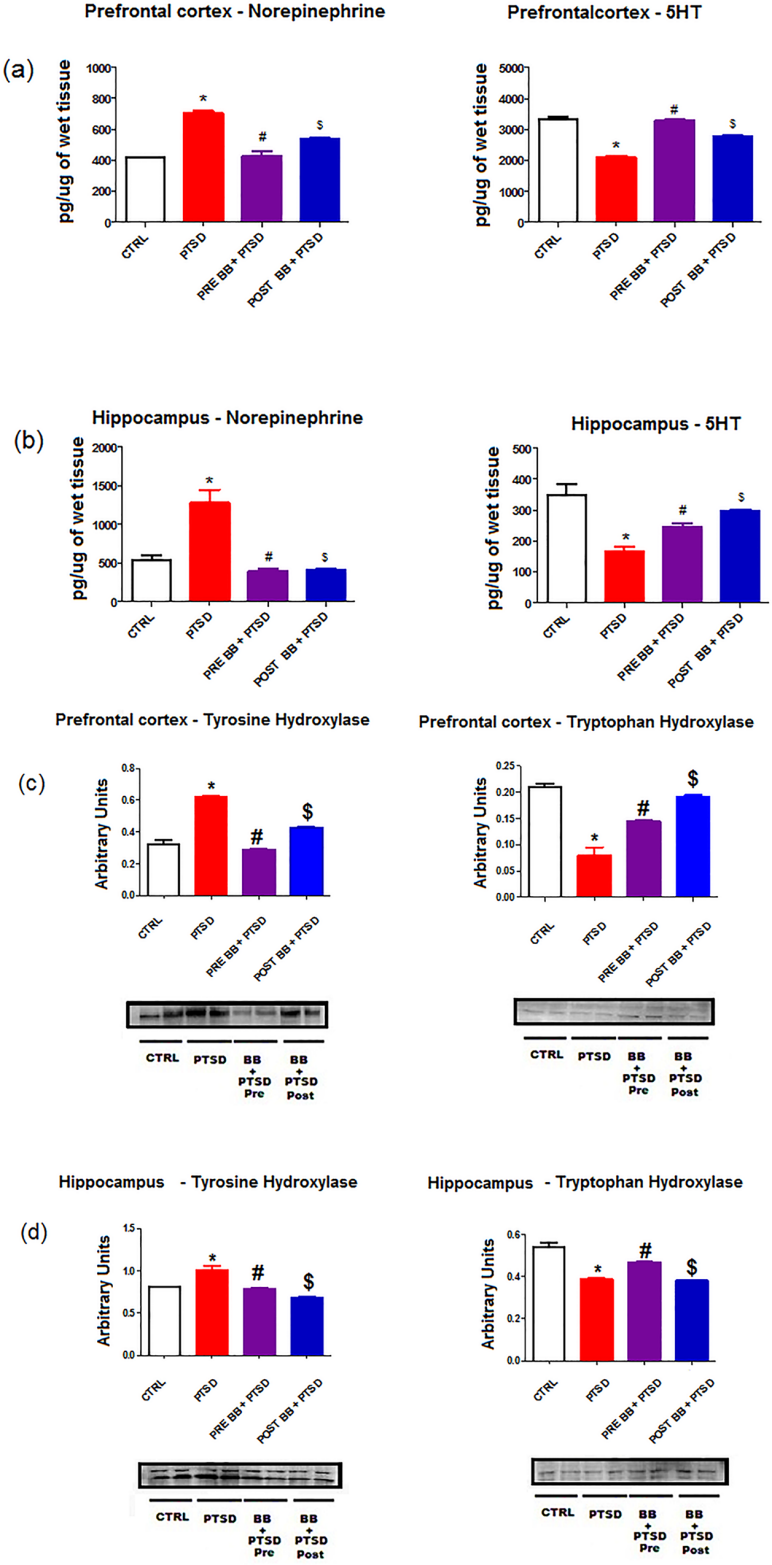
a and b: Neurotransmitters quantification: In the PFC and Hippocampus region the NE levels were significantly higher in the PTSD group Vs controls (p<0.05). On the contrary the monoamine 5-HT levels were significantly lower in the PTSD group Vs control. Blueberry enriched diet lowered NE levels and increased 5-HT levels similar as the untreated controls. Statistical analysis was performed using one way ANOVA with repeated measurements followed by Tukey’s multiple comparison test. *p<0.05 Control Vs PTSD, #p<0.05 PTSD Vs BB = PTSD, and $p<0.05 PTSD Vs PTSD + BB. All data are presented as mean ± SEM. P<0.05 compared to control. c and d: Rate-limiting enzymes tyrosine hydroxylase (catecholamines) and tryptophan hydroxylase (5-HT) post stress: Tyrosine hydroxylase elevation in the PFC and hippocampus corroborated the findings of elevated NE levels. The elevation was normalized to control group levels with blueberry diet. The tryptophan hydroxylase levels were downregulated in the PFC and hippocampus. The downregulation was upregulated with blueberry diet. Statistical analysis was performed using one way ANOVA with repeated measurements followed by Tukey’s multiple comparison test. *p<0.05 Control Vs PTSD, #p<0.05 PTSD Vs BB = PTSD, and $p<0.05 PTSD Vs PTSD + BB. All data are presented as mean ± SEM. P<0.05 compared to control.

In the PFC and hippocampus of the PTSD group the norepinephrine levels were significantly elevated when compared with control (p < 0.05). The levels were significantly (p < 0.05) diminished in the animals fed with 2% blueberry diet. The group with pre blueberry diet + PTSD Vs PTSD showed more effect than post blueberry diet + PTSD (p < 0.05). In contrast, the 5-HT levels were significantly reduced in the PTSD group when compared with control. In PFC the 5-HT levels were elevated with pre blueberry diet + PTSD Vs PTSD displayed more effect than post blueberry diet + PTSD (p < 0.05). Interestingly, in Hippocampus the 5-HT levels were elevated with post blueberry diet + PTSD Vs PTSD displayed more effect than pre blueberry diet + PTSD (p < 0.05) when compared with control. Blueberry diet increased 5-HT levels without increasing the norepinephrine levels (p < 0.05) of the experimental animals (Fig 4A and 4B).

### Rate-limiting enzymes quantification

To verify the detected changes in neurotransmitters levels in the PFC and hippocampus, we did Western Blots analysis for the rate limiting enzymes for norepinephrine [(tyrosine hydroxylase (TH)] and 5-HT [(tryptophan hydroxylase (TPH)]. In PFC and hippocampus of the PTSD animals, the TH levels were significantly (p < 0.05) elevated when compared with control and it diminished in the animals fed with 2% blueberry diet (p < 0.05). Interestingly, in PFC the TH levels were elevated with post blueberry diet + PTSD Vs PTSD displayed more effect than pre blueberry diet + PTSD (p < 0.05) when compared with control. In contrast, in hippocampus the TPH levels were diminished with post blueberry diet + PTSD Vs PTSD displayed more effect than pre blueberry diet + PTSD (p < 0.05) when compared with control(Fig 4C and 4D).

### Inflammatory markers

**Fig 5:**
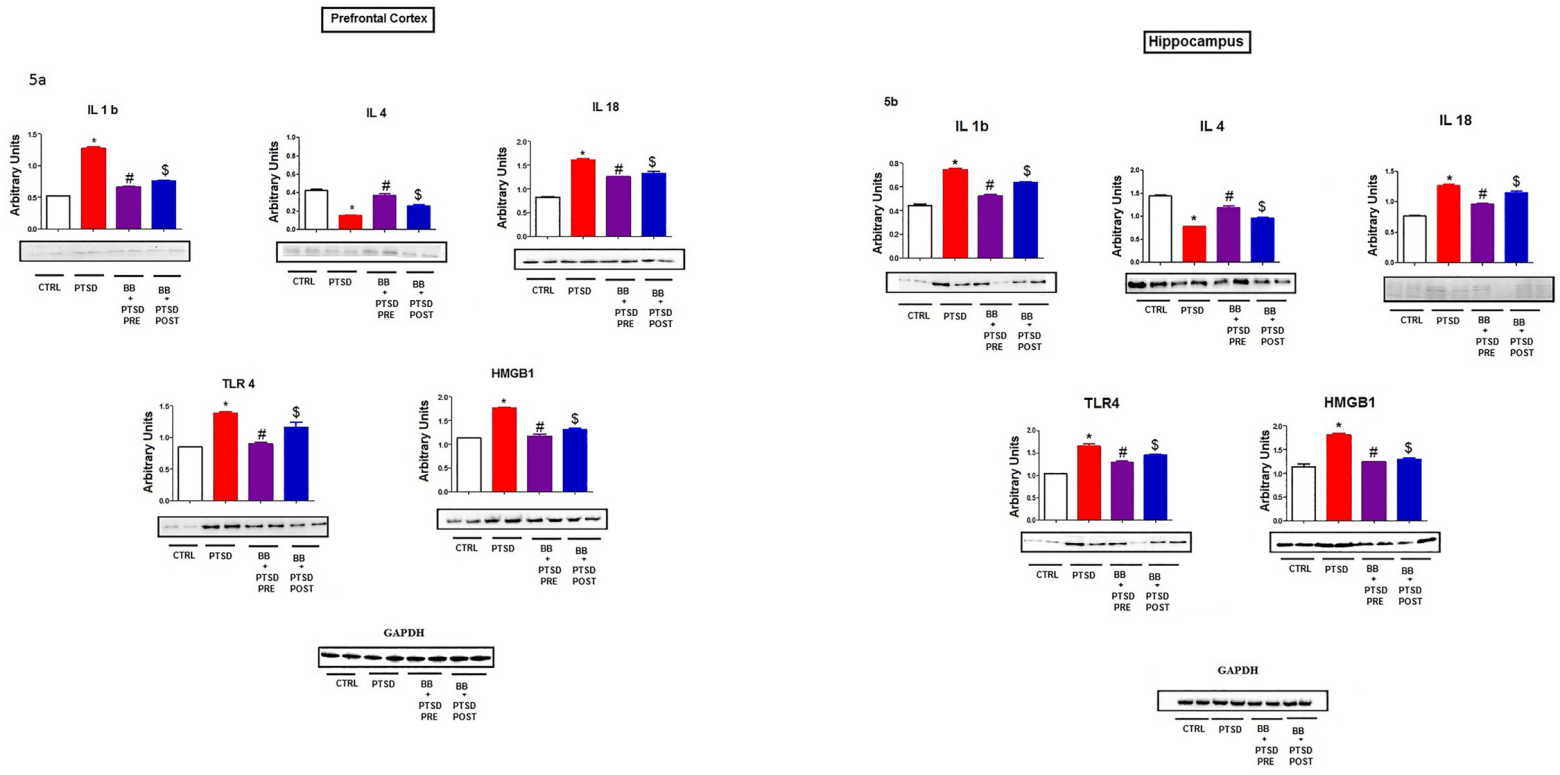
a: Pro and anti-inflammatory Marker Protein: The prefrontal cortex of the PTSD group showed elevated levels (p<0.05) of IL-1b, IL18, TLR4 and HMGB1. In contrast, the level of anti-inflammatory protein IL-4 were lower (p < 0.05) in the PTSD group. Blueberry enriched diet shows the significant reduction in the levels of proinflammatory cytokines (IL-1b, IL-18) in addition to TLR4 and HMGB1. Statistical analysis was performed using one way ANOVA with repeated measurements followed by Tukey’s multiple comparison test. *p<0.05 Control Vs PTSD, #p<0.05 PTSD Vs BB = PTSD, and $p<0.05 PTSD Vs PTSD + BB. All data are presented as mean ± SEM. P<0.05 compared to control. b: Pro and anti-inflammatory Marker Protein: The Hippocampus of the PTSD group showed elevated levels (p<0.05) of IL-1b, IL18, TLR4 and HMGB1. In contrast, the level of anti-inflammatory protein IL-4 were lower (p < 0.05) in the PTSD group. Blueberry enriched diet shows the significant reduction in the levels of proinflammatory cytokines (IL-1b, IL-18) in addition to TLR4 and HMGB1. Statistical analysis was performed using one way ANOVA with repeated measurements followed by Tukey’s multiple comparison test. *p<0.05 Control Vs PTSD, #p<0.05 PTSD Vs BB = PTSD, and $p<0.05 PTSD Vs PTSD + BB. All data are presented as mean ± SEM. P<0.05 compared to control.

**Fig 6:**
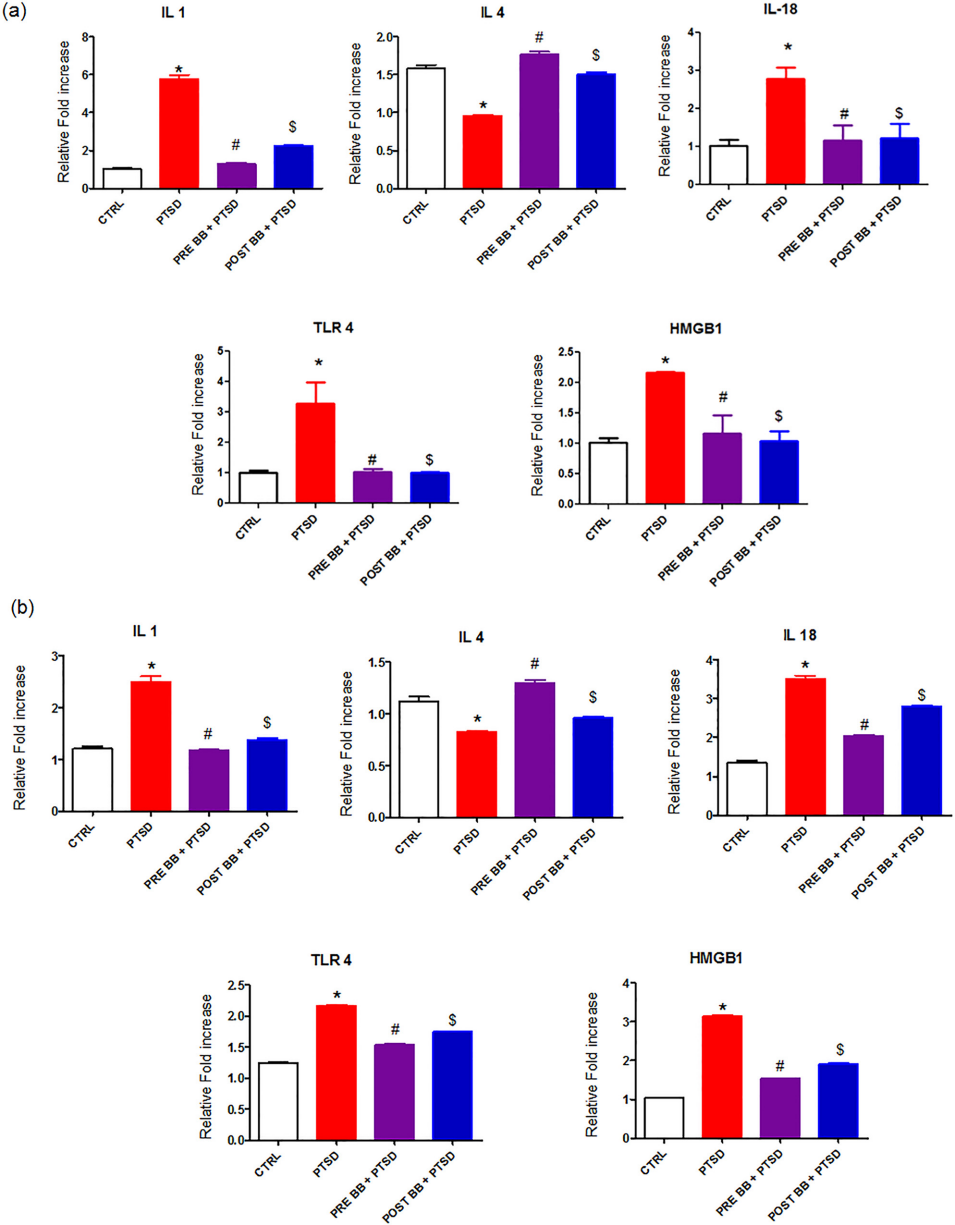
a: RT-PCR mRNA: The prefrontal cortex region of the PTSD group showed elevated levels (p<0.05) of IL-1b, IL-18, TLR4 and HMGB1. In contrast, the level of anti-inflammatory protein IL-4 were lower (p<0.05) in the PTSD group. Blueberry enriched diet shows the significant reduction in the levels of inflammatory markers. Statistical analysis was performed using one way ANOVA with repeated measurements followed by Tukey’s multiple comparison test. *p<0.05 Control Vs PTSD, #p<0.05 PTSD Vs BB = PTSD, and $p<0.05 PTSD Vs PTSD + BB. All data are presented as mean ± SEM. P<0.05 compared to control. b: RT-PCR mRNA: The Hippocampus region of the PTSD group showed elevated levels (p<0.05) of IL-1b, IL-18, TLR4 and HMGB1. In contrast, the level of anti-inflammatory protein IL-4 were lower (p<0.05) in the PTSD group. Blueberry enriched diet shows the significant reduction in the levels of inflammatory markers. Statistical analysis was performed using one way ANOVA with repeated measurements followed by Tukey’s multiple comparison test. *p<0.05 Control Vs PTSD, #p<0.05 PTSD Vs BB = PTSD, and $p<0.05 PTSD Vs PTSD + BB. All data are presented as mean ± SEM. P<0.05 compared to control.

In the PFC and hippocampus, the PTSD group showed an elevated level of IL-1 IL18, TLR4 and HMGB1 (p < 0.05) when compared to controls and it diminished in the animals fed with 2% blueberry diet (p < 0.05). Interestingly, the level of anti-inflammatory protein IL-4 were lower (p < 0.05) in the PTSD group. Blueberry enriched diet shows the significant reduction in the levels of proinflammatory cytokines (IL-1b, IL-18) in addition to TLR4 and HMGB1. In IL-4, the group with pre blueberry diet + PTSD Vs PTSD showed more effect when compared to post blueberry diet + PTSD (p < 0.05) group when compared with control. Interestingly, in TLR4, the group with post blueberry diet + PTSD Vs PTSD showed more effect when compared to pre blueberry diet + PTSD (p < 0.05) group (Fig 5A and 5B) when compared with control. Blueberry diet normalized the levels of proinflammatory cytokines mRNA and upregulated anti-inflammatory cytokine levels (Fig 6A and 6B).

## Discussion

Reactive oxygen species (ROS) encompass a variety of diverse chemical species including superoxide anions, hydroxyl radicals, and hydrogen peroxide, which are freely diffusible and relatively long-lived [33–36]. These ROS (Fig 1B) are transported to several tissues through the blood stream and trigger the acute phase response which leads to the significant generation of free radicals in tissues [37, 38]. Our results show an increase in total ROS production in the prefrontal cortex and hippocampus of the PTSD animals and a decrease in the levels of ROS in the animals fed with 2% blueberry diet (Fig 2A & 2B). This finding suggests that the antioxidant components in the blueberry diet play a major role in the cellular defense mechanisms and protect against oxidative damage by scavenging the free radicals. Since blueberries are rich sources of anthocyanin polyphenols, they can act as antioxidant and anti-inflammatory molecules and confer considerable potential protective effects against development of inflammation related diseases [14, 17, 19–21].

Hyperarousal and intensified fretfulness lead to diminished cognition [5, 39]. It is one of the diagnostic categories of PTSD [40, 41]. Earlier investigation through anxiety testing with a similar predator exposure model also confirmed memory-related changes of the hippocampus [5]. To measure anxiety levels, we used the elevated plus-maze (EPM). The experimental rats have the natural tendency to explore new surroundings, but open zones or lanes invoke a greater fear and evasion response. The EPM is widely used as a measure to test fear or anxiety and has been extensively validated for use in rats [10–12]. The principle behind the EPM is based on rodents' natural aversion of open spaces versus their desire to explore new surroundings. Entry into the closed circuit is related with amplified freezing behavior as well as elevated plasma corticosterone levels, representing keen anxiety. Anxiolytic agents can increase evasion of the fear-provoking open arms while anxiolytic agents can elevate open arm exploration [42, 43]. Our results show that the PTSD animals exhibited increased anxiety compared to the control group (Fig 3), as evidenced by their reluctance to spend time in open arms of the EPM.

Neurotransmitters and neuropeptides from the central nervous system are known to influence the functions of the immune system, but the extent of their contribution in the enhancement of immune reactivity needs to be examined in inflammation associated with PTSD [9]. The future prospects in these lines might elucidate the significance of these neurochemical changes on neural circuitry underlying the central effects of the immune system [44, 45]. Our present findings reveal that the supplementation of a blueberry diet would represent a viable therapeutic approach to diminish oxidative vulnerability in the central nervous system and modulate the levels of neurotransmitters in the hippocampus and neocortex regions. Our results show that (Fig 4A–4D) the dysfunction in monoaminergic metabolism of PTSD animals is not only due to loss of synapses of particular neurons, but is also caused by dysfunction in biosynthesis of monoaminergic neurotransmitters. We have measured NE and 5-HT in the prefrontal cortex and hippocampus regions. In our previous study [10] we have shown that the PTSD animals treated with Sertraline (SSRI) had significant increases in 5-HT levels in the prefrontal cortex and hippocampus regions and cerebrospinal fluid (CSF) in addition to NE levels and it may enhance the PTSD pathophysiology. Our results show that the blueberries increase the 5-HT levels without increasing NE levels (Fig 4A and 4B).

Moreover, we also measured the rate limiting enzymes tyrosine hydroxylase and tryptophan hydroxylase. These enzymes play a key role in the catabolism of neurotransmitters [9, 41]. Our results show that in PTSD animals the levels of tyrosine hydroxylase were elevated and subsequently reduced with the blueberry diet. In contrast, the tryptophan hydroxylase levels were reduced in the PTSD group and were increased with the blueberry diet (Fig 4C and 4D). It has been well known that neuroinflammation characterized by microglial activation may tangle in the degeneration of dopaminergic neurons in neurological disorders [46, 47]. Microglia may release an extensive range of proinflammatory cytokines that can affect neuronal circuitry and may cause an imbalance in the neurotransmitters, thereby intensifying the anxiety [48].

PTSD activates inflammatory cells to generate a variety of proinflammatory and cytotoxic factors, including the proinflammatory cytokines like (Interleukin) IL1, and IL 18 [10–12]. Our result showed that proinflammatory cytokines may mediate neuronal degeneration through the activation of a cascade of inflammatory factors following subsequent increased release of ROS from the neuronal cells (Fig 2A and 2B). The anti-inflammatory cytokine IL4 levels were decreased in the PTSD group and alleviated with the blueberry diet. In addition to proinflammatory (PIC) and anti-inflammatory cytokines, we also measured the levels of TLR4 and HMGB1. The interaction of TLR4 and HMGB1 resulted in the upregulation of NF-κB which contributes to the production and release of proinflammatory cytokines (Fig 5A and 5B) and may also contribute to the generation of ROS (Fig 2A & 2B). Earlier studies show that the activation of TLRs leads to the synthesis of pro-inflammatory cytokines and chemokines and the expression of co-stimulatory molecules [49, 50]. Our animal model of PTSD offers an understanding of a possible mechanistic relationship between physiological stress and oxidative stress. There is a large body of pre-clinical literature describing mechanisms of inflammation and oxidative stress [51, 52]. Earlier human PTSD studies show that there is a significantly higher release of proinflammatory cytokine IL6 concentrations [53, 54]. Our results show that (Figs 5A, 5B, 6A & 6B) feeding blueberries prevents the enhanced levels of PICs IL1, IL4 and IL 18 in addition to TLR4 and HMGB1, thereby preventing the catabolism of biogenic amines [55, 56]. HMGB1 is an essential cytokine that mediates the response to infection, injury and inflammation [57, 58]. The occurrence of HMGB1 in the nucleus is governed by posttranslational modifications of the cellular reactions. Once the protein is not acetylated, it stays in side of the nucleus, but during inflammation the hyperacetylation occurs on lysine residues causing it to translocate into the cytosol. Together, our results clearly reveal the links between PTSD induced oxidative stress and the protective effects of blueberries.

In summary, our results suggest a major role of blueberries in protecting against inflammation associated with PTSD through a decrease in the levels of inflammatory cytokines and by scavenging free radicals. This leads to significant downregulation of total ROS, resulting in anti-inflammatory action by increasing 5-HT levels without increasing NE levels. In addition, blueberries appear to enhance resiliency behavior as indicated by reduced anxiety levels. Therefore, it can be concluded that nonpharmacological approaches using blueberries might be a useful addition for its anti-inflammatory action in the animal model of PTSD.
